# Redescription and redefinition of the genus *Chiltana* Shakila-Mushtaq & Akbar, 1995 (Hemiptera, Fulgoromorpha, Dictyopharidae, Dictyopharini), with description of a new species from Pakistan

**DOI:** 10.3897/zookeys.838.30910

**Published:** 2019-04-11

**Authors:** Zhi-Shun Song, Imran Khatri, Ai-Ping Liang

**Affiliations:** 1 Jiangsu Key Laboratory of Biofunctional Molecules, School of Life Sciences, Chemistry & Chemical Engineering,; 2 Jiangsu Second Normal University, Nanjing, China; 3 Department of Entomology, Sindh Agriculture University, Tandojam, Pakistan; 4 Key Laboratory of Zoological Systematics and Evolution,; 5 Institute of Zoology, Chinese Academy of Sciences, Beijing, China

**Keywords:** Fulgoroidea, morphology, taxonomy

## Abstract

The genus *Chiltana* Shakila-Mushtaq & Akbar, 1995 is redescribed and redefined based on the types and new material from Pakistan. *Chiltana* includes two species, *C.acarinata***sp. n.** and *C.baluchi* Shakila-Mushtaq & Akbar, 1995 (the type species), both from Chiltan, Balochistan, Pakistan. A key to the species of the genus is provided. Nomenclatorial remarks on original publication, author, and date of *Chiltana* are given.

## Introduction

The dictyopharid planthopper genus *Chiltana* was firstly described by Shakila-Mushtaq in her Ph.D. thesis for a single species from Pakistan (Shakila-Mushtaq 1984). Shakila-Mushtaq (1994) listed this genus in her paper “Family Dictyopharidae (Fulgoroidea: Homoptera) from Pakistan” and stated that “*Chiltana*, a new monotypic genus has been described from Pakistan by Shakila-Mushtaq (1989 [sic]) to be added in the family Dictyopharidae” (Shakila-Mushtaq 1994: 30). One year later, Shakila-Mushtaq and Akbar (1995) erected *Chiltana* as a new genus formally published in a peer-reviewed journal. Thus the original publication, author and date of *Chiltana* have been debatable.

*Chiltana* was placed in the tribe Dictyopharini (Emeljanov 2011). The morphological phylogeny of the world Dictyopharidae showed that *Chiltana* is quite unique in Dictyopharini and has many autapomorphies supporting its monophyly but affecting its phylogenetic assessment in the tribe Dictyopharini (Song et al. 2018).

Based on examination of the type specimens of *C.baluchi* and a critical review of the literature, *Chiltana* is here redescribed and redefined. The second *Chiltana* species, *C.acarinata* sp. n., is described and illustrated from Pakistan. Nomenclatorial remarks on original publication, author, and date of *Chiltana* are given.

## Material and methods

The specimens studied in the course of this work are deposited in the following institutions, which are subsequently referred to by their acronyms: CAS, California Academy of Sciences, San Francisco, CA, USA; and ZMUK, Zoological Museum, University of Karachi, Karachi, Pakistan.

The post-abdomen of the specimens used for dissections were cleared in 10% KOH at room temperature for ca 6–12 hours, rinsed and examined in distilled water and then transferred to 10% glycerol and enclosed in microvials to be preserved with the specimens. Observations and measurements were conducted under a Zeiss Stemi SV II optical stereomicroscope, and photography was under Zeiss Discovery V12 stereomicroscope equipped with a Nikon D7000 digital camera in Institute of Zoology, Chinese Academy of Sciences, China. Some final images were compiled from multiple photographs using CombineZM image stacking software and improved with the Adobe Photoshop CS5 software.

The morphological terminology and measurements used in this study follow Song et al. (2016a, b, 2018) for most characters and Bourgoin et al. (2015) for the forewing.

## Taxonomy

### Family Dictyopharidae Spinola, 1839

#### Genus Chiltana

Taxon classificationAnimaliaHemipteraDictyopharidae

Shakila-Mushtaq & Akbar, 1995

Chiltana Shakila-Mushtaq, 1984: 158, nomen nudum of Chiltana Shakila-Mushtaq & Akbar, 1995.
Chiltana Shakila-Mushtaq, 1994: 2 (in key), 30 (in catalogue), nomen nudum of Chiltana Shakila-Mushtaq & Akbar, 1995.
Chiltana Shakila-Mushtaq & Akbar, 1995: 374. Type species: Chiltanabaluchi Shakila-Mushtaq & Akbar, 1995; by original designation and monotypy.

##### Diagnosis.

*Chiltana* may be distinguished from other genera in the Dictyopharini by the following combination of characters: cephalic process absent due to anterior margin of vertex not reaching beyond anterior margin of eyes; vertex with lateral carinae weakly ridged and subparallel, anterior and posterior margins nearly straight, without median carina; frons without median and intermediate carinae; pronotum with anterior and posterior margins nearly straight and subparallel anteroposteriorly, lateral marginal areas distinctly convex, median carina complete but weak, without intermediate carinae; mesonotum distinctly arched, carina absent; fore and middle femora flattened and dilated, with several various sized spines on ventral margin; hind tibiae with eight apical teeth; phallobase with inflated membranous paired lobes, with numerous small superficial spines on apex of lobes.

##### Redescription.

Head very short, cephalic process absent due to anterior margin of vertex not reaching beyond anterior margin of eyes, so anterior part of dorsal surface of head occupied by basal extension of frons in dorsal view (Fig. 2A). Vertex (Fig. 2A) moderately broad, basal width slightly wider than transverse diameter of eyes; anterior margin not reaching beyond anterior margin of eyes, just approaching apical fourth of eyes; posterior plane elevated above pronotum; lateral carinae weakly ridged and subparallel; anterior and posterior margins weakly ridged and nearly straight; median carina absent, with a lateral large pit on each side. Frons (Fig. 2C) with lateral carinae weakly ridged, nearly parallel; median and intermediate carinae absent; basal margin of frons projecting anteriad to apex of vertex, distinctly visible in dorsal view (Fig. 2A). Postclypeus and anteclypeus (Fig. 2C) convex medially, with distinct median carina. Rostrum moderately long, reaching base of hind femora; basal segment slightly longer than distal one. Compound eyes large and globose. Ocelli relatively large, reddish. Antennae with very small scape; pedicel large and subglobular, with more than 50 distinct sensory plaque organs distributed over entire surface; flagellum long, setuliform.

Pronotum (Fig. 2A) distinctly shorter than mesonotum at midline, anterior and posterior margins nearly straight and subparallel anteroposteriorly; lateral marginal areas distinctly convex and sloping down with two longitudinal carinae on each side; intermediate carinae absent; median carina complete but weak, with a lateral pit on each side. Mesonotum (Fig. 2A) distinctly arched, carina absent. Forewings (Fig. 2D) hyaline, venation with sparse transverse veins; MP bifurcating MP_1+2_ and MP_3+4_ near middle and beyond CuA; number of apical cells between R and CuA equal to 13; Pcu and A_1_ veins fused into a long Pcu+A1 vein at apical 1/4 in clavus; pterostigmal area clear, with 4 or 5 cells. Legs (Fig. 3A–D) moderately long; fore femora strongly flattened and dilated, with about 10 various sized spines on ventral margin; middle femora flattened and dilated, with about six various sized spines on ventral margin; fore and middle tarsomeres I and II with several acutellae; hind tibiae with four lateral spines and eight apical teeth; hind tarsomeres I and II with about 14 apical teeth, respectively.

Male genitalia. Pygofer (Fig. 4A, B, D) in lateral view wider ventrally than dorsally, dorsal margin slightly excavated to accommodate segment X, dorsoposterior margins angular, produced into a distinct lobe which is short and broad. Gonostyles (Fig. 4B–E) symmetrical, with narrow base, expanded toward apex, broadest at apical fourth; dorsal margin with a claw-like process directed dorsad, outer dorsal edge with a spiny hook-like process near middle directed ventrad. Aedeagus (Fig. 5A–F) with one pair of elongate endosomal processes extended from phallobase membranous, acute and sclerotised apically; phallobase sclerotised and pigmented basally, membranous and inflated apically, with paired lobes. Segment X (Fig. 4A, B) large, in dorsal view, with apex deeply excavated to accommodate anal style; anal style elongate and large.

Female genitalia. Gonapophyses VIII with anterior connective lamina large and sclerotized, with teeth of varying sizes and shapes. Gonoplacs with two lobes homologous; lateral lobe sclerotized, large and elongate, with a distinct sensory appendage on apex, a bunch of long setae on sensory appendage; the posterior lobe membranous, containing long sclerotized plate. Segment X large and broad, apex deeply excavated to accommodate anal style; anal style large and elongate.

##### Diversity and distribution.

This genus contains two species restricted to Chiltan, Balochistan, Pakistan.

##### Remarks.

In the original descriptions and illustrations of *Chiltana*, the frons and mesonotum were described as “tricarinate” (Shakila-Mushtaq and Akbar 1995). Actually, the carinae on the frons and mesonotum of *Chiltana* species are absent based on examination of the type specimens of *C.baluchi* and new species, although these corresponding positions show the different colored patterns, like some other dictyopharid species. In addition, the legs, female genitalia and other characters of *Chiltana* were not mentioned in the original paper. The original generic diagnosis of *Chiltana* is short and incomplete. Thus, *Chiltana* is here redescribed and redefined based on examination of the type specimens of *C.baluchi* and new species and a critical review of the literature.

*Chiltana* is similar to the genera *Afronersia* Fennah, 1958 and *Gilgitia* Shakita-Mushtaq, 1991 in various characters of head, venation and genitalia (Shakila-Mushtaq and Akbar 1995). In the tribe Dictyopharini, *Chiltana* has several diagnostic characters that serve to differentiate it from other genera. The smaller dimensions of the head, absence of carinae on the frons and mesonotum, and flattened and dilated fore and middle femora with variously sized spines on the ventral margin may easily distinguish *Chiltana* from remaining genera in the Dictyopharini.

### Key to the species of *Chiltana*

**Table:** 

1	Aedeagus with endosomal processes directed ventrad; dorsal apical process of gonostyles large and broad, directed dorsad; phallobase with a pair of dorsal lobes with a large and stout spine on apex of each lobe	***C.acarinata* sp. n.**
–	Aedeagus with endosomal processes directed dorsad; dorsal apical process of gonostyles small, directed dorsocephalad; phallobase with two pairs of dorsal lobes, without spine on apex	***C.baluchi* Shakila-Mushtaq & Akbar**

#### Chiltana acarinata sp. n.

Taxon classificationAnimaliaHemipteraDictyopharidae

http://zoobank.org/40014A57-8B37-4C06-832D-2599286E843D

**Figure 1.**
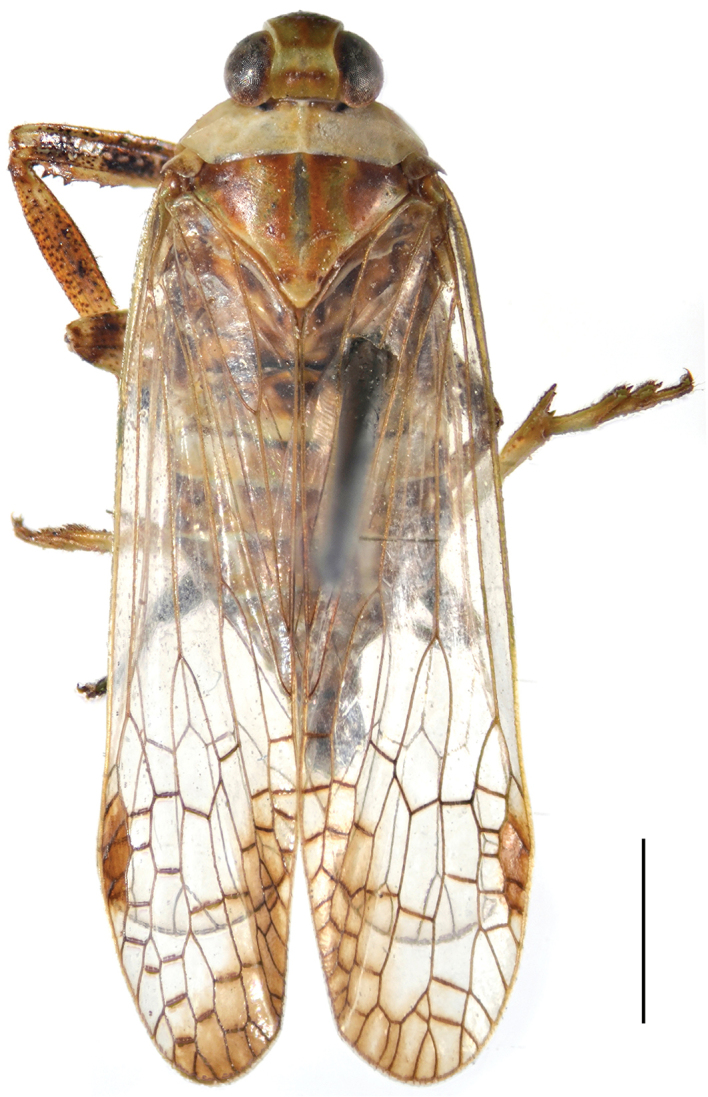
Habitus of *Chiltanaacarinata* sp. n. Scale bar: 2 mm.

**Figure 2.**
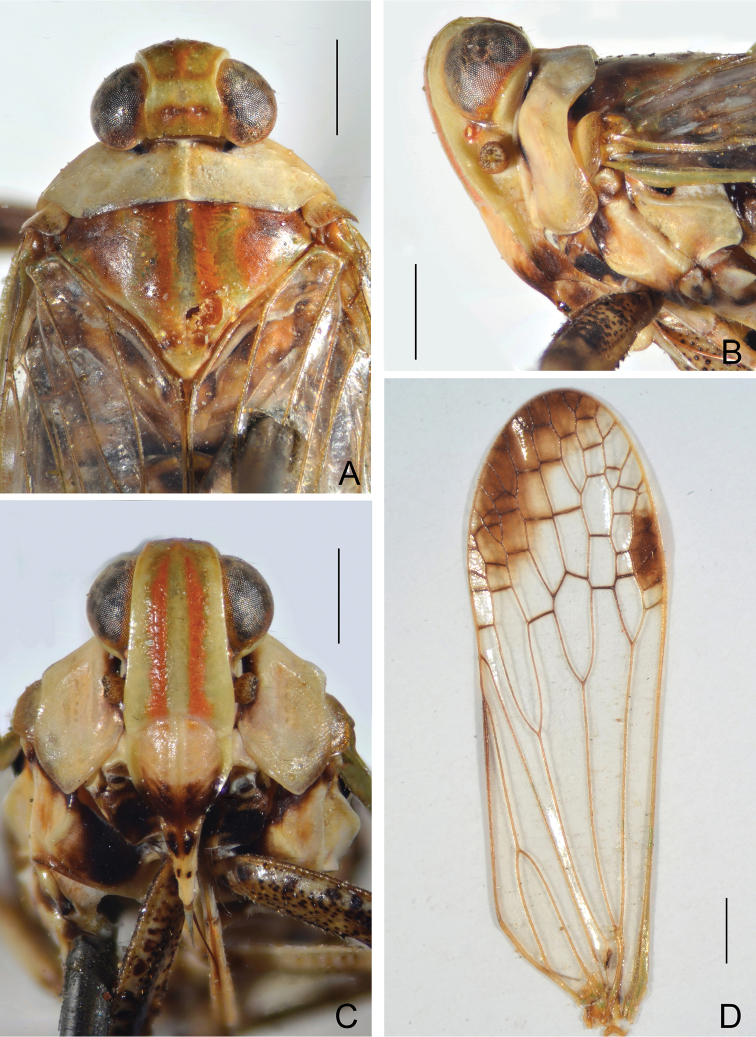
*Chiltanaacarinata* sp. n. **A** Head, pronotum and mesonotum, dorsal view **B** head and pronotum, lateral view **C** head and pronotum, ventral view **D** forewing. Scale bars: 1 mm.

**Figure 3.**
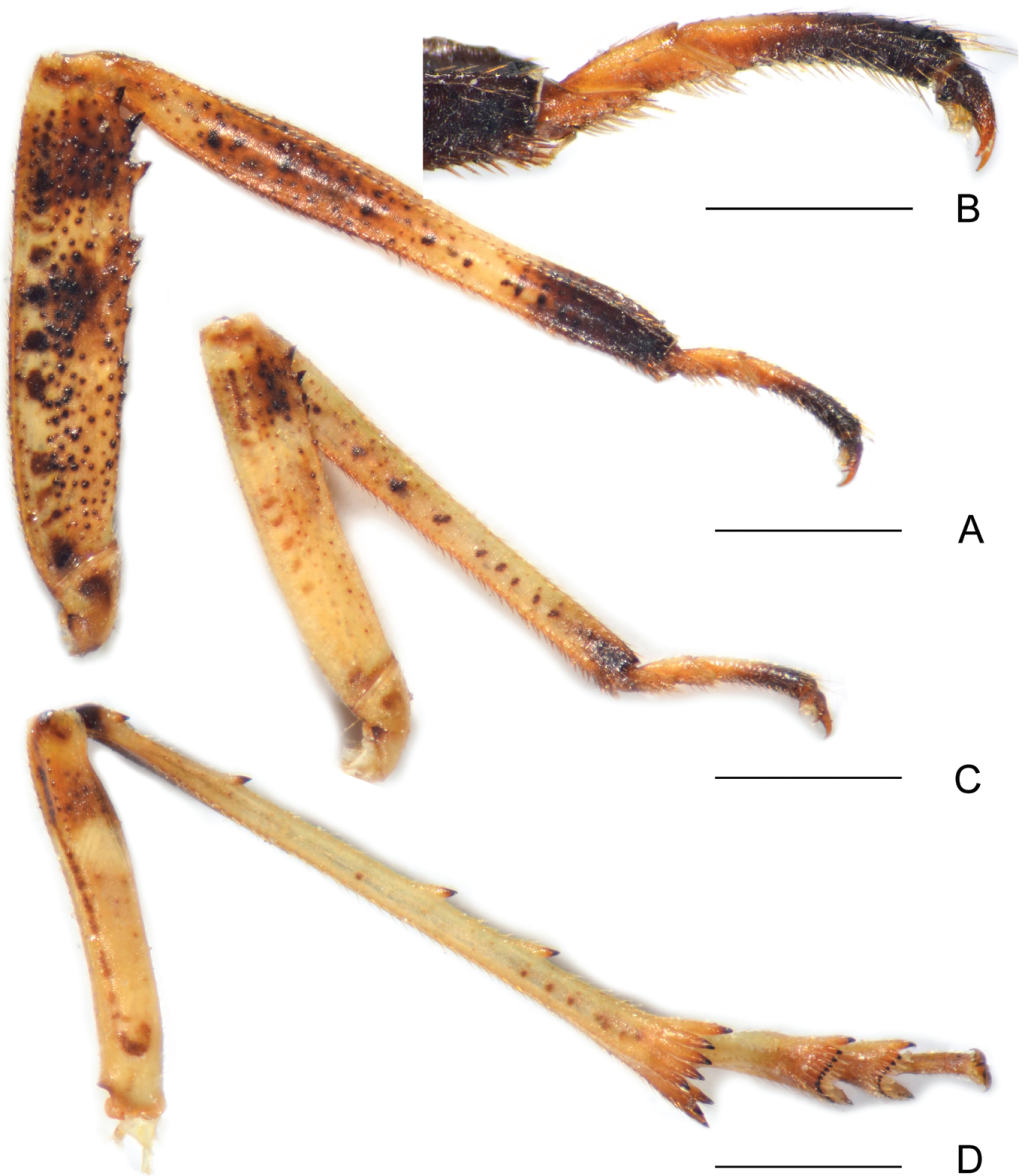
*Chiltanaacarinata* sp. n. **A** Fore leg **B** fore tarsomeres and pretarsus **C** middle leg **D** hind leg. Scale bars: 1 mm.

**Figure 4.**
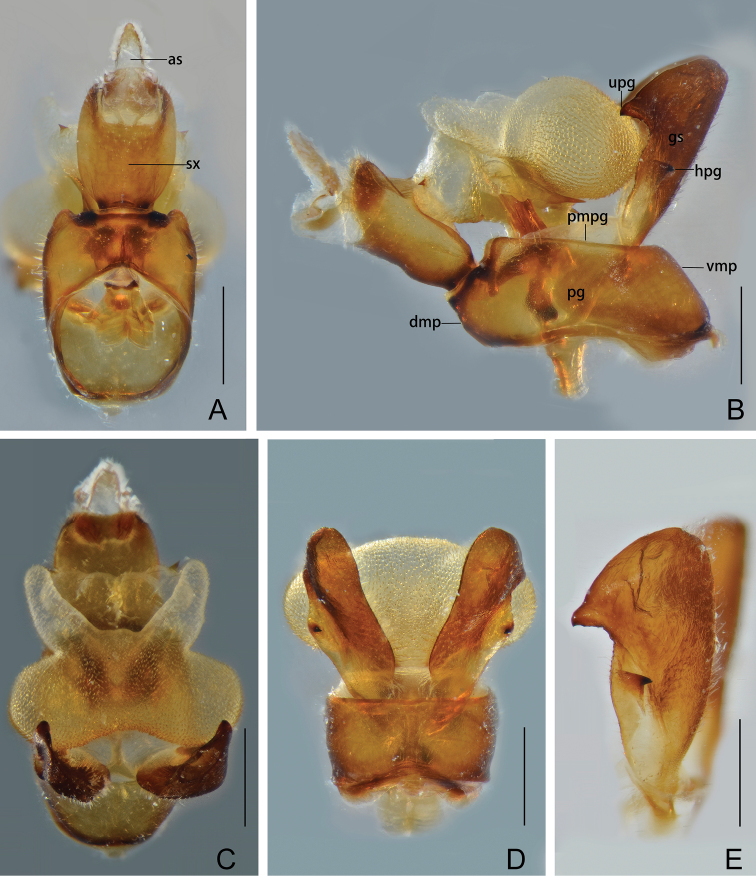
*Chiltanaacarinata* sp. n. **A** Male segment X and pygofer, dorsal view **B** male pygofer, gonostyles, and segment X, lateral view **C** male pygofer, gonostyles, and segment X, caudal view **D** male pygofer and gonostyles, ventral view **E** gonostyle. Abbreviations: as, anal style; dmp, dorsal margin of pygofer in profile; gs, gonostyle; hpg, hook-like process of gonostyle; pg, pygofer; upg, upper process of gonostyle; sx, segment X; vmp, ventral margin of pygofer in profile. Scale bars: 0.5 mm.

**Figure 5.**
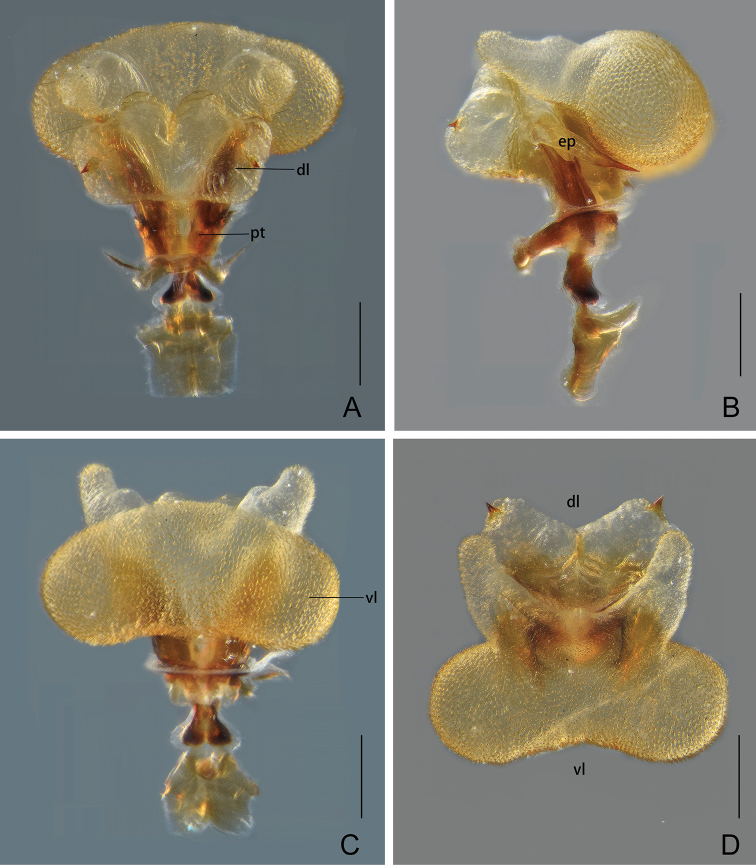
*Chiltanaacarinata* sp. n. **A** Aedeagus, dorsal view **B** aedeagus, lateral view **C** aedeagus, ventral view **D** aedeagus, caudal view. Abbreviations: dl, dorsolateral lobe of phallotheca; ep, endosomal processes; pt, phallotheca; vl, ventral lobe of phallotheca. Scale bars: 0.2 mm.

Figures 1 , 2 , 3 , 4 , 5

##### Type material examined.

Holotype ♂, Pakistan: Hazarganji, Chiltan National Park, 20 km SW Quetta, 3–6.vii.1989, W.J. Pulawski & W.A. Khan (CAS). Paratype, 1 ♂, Pakistan: same as holotype (CAS)

##### Description.

Body length (from apex of head to tip of forewings): 11.5–11.7 mm; head length (from apex of head to base of eyes): 1.2 mm; head width (including eyes): 1.5 mm; forewing length: 9.6–9.7 mm.

Coloration. General color brownish ochraceous marked with ivory white, pale green and purplish red on head and thorax, and dark brown on abdomen in dorsal view (Fig. 1). Head excluding eyes ivory white, vertex ochraceous basally and yellowish green apically (Fig. 2A), frons yellowish green, areas along intermediate carinae purplish red (Fig. 2C). Compound eyes fuscous with posterior margin ochraceous red and ivory white, ocelli purplish red (Fig. 2B). Clypeus pale ochraceous basally and apically, and dark brown medially, with a pair of small black spots on anteclypeus (Fig. 2C). Pronotum entirely ivory white. Mesonotum purplish red to ochraceous brown, areas of median and lateral carinae and lateral marginal areas flavescent or greenish (Fig. 2A). Forewings membrane hyaline, veins ochraceous, pterostigmal area and a large sublunate streak on distal fourth dull ochraceous (Fig. 2D). Thorax yellowish ochraceous ventrolaterally with dark brown patches adjacent to base of fore coxae. Legs pale to dark brown, with numerous black small spots (Fig. 3A–D). Abdomen dorsally ochraceous to dark brown, with dark brown or pale ochraceous stripes of various sizes and shape, ventrally more or less uniformly yellowish ochraceous; male and female terminalia brown.

Structure. Head (Fig. 2A–C) very short, cephalic process absent. Vertex (Fig. 2A) wider than length, with ratio of length at midline to width between eyes 0.8:1. Frons with basal margin of frons projecting anteriad to apex of vertex, distinctly visible in dorsal view (Fig. 2A); in ventral view, frons with ratio of length at midline to maximum width 2.0:1; median and intermediate carinae absent (Fig. 2C). Forewings (Fig. 2D) hyaline, ratio of length to width about 3.2:1. Legs (Fig. 3A–D) moderately long; fore femora (Fig. 3A) strongly flattened and dilated, with about 10 various sized spines on ventral margin; middle femora (Fig. 3C) flattened and dilated, with about six variously sized spines on ventral margin; fore and middle tarsomeres I and II (Fig. 3B) with several acutellae; hind tibiae (Fig. 3D) with four lateral spines and eight apical teeth; hind tarsomeres I and II with about 14 apical teeth, respectively.

Male genitalia. Pygofer, in lateral view (Fig. 4B), with dorsoposterior margin forming a small and broad lobe; in ventral view (Fig. 4D) a little longer than in dorsal view (Fig. 4A) with ratio of ventral to dorsal width about 1.3:1. Gonostyles (Fig. 4B–E) elongate, relatively narrow in basal half, dorsal apical process large and broad, directed dorsad (Fig. 4E). Aedeagus (Fig. 5A–C) large and strongly inflated, endosomal processes elongate and robust, extended from phallobase, curved dorsad and then ventrad, apex sclerotized, elongate and acute (Fig. 5B). Phallobase with three pairs of inflated membranous lobes: a pair of large and stout dorsal lobes, directed dorsad, with a large and stout spine on apex of each lobe (Fig. 5A, B, D); a pair of large, strongly inflated, rounded ventral lobes, directed laterad, covered with numerous minute superficial spines (Fig. 5B–D); and a pair of elongate thumb-like ventral lobes extended from dorsal side of rounded ventral lobes, their apices gradually convergent and tapering dorsad, muricate apically (Fig. 5B–D). Segment X, in dorsal view (Fig. 5A), oval and broadest medially, with ratio of length to maximum width 1.1:1; in lateral view (Fig. 5B), short and robust, with ventral margin gradually widening from base to apex; anal style large, beyond apical ventral margin of segment X.

##### Etymology.

The specific epithet is borrowed from New Latin *acarinatus*, referring to the carinae on the frons and mesonotum being absent.

##### Distribution.

So far only known from Chiltan, Balochistan, Pakistan.

##### Remarks.

The new species may be distinguished from the type species of *Chiltana*, *C.baluchi*, by the different male genitalia.

#### Chiltana baluchi

Taxon classificationAnimaliaHemipteraDictyopharidae

Shakila-Mushtaq & Akbar, 1995

Chiltana baluchi Shakila-Mushtaq, 1984: 160, fig. 33A–I, nomen nudum of Chiltanabaluchi Shakila-Mushtaq & Akbar, 1995.
Chiltana baluchi Shakila-Mushtaq, 1994: 30 (in catalogue), nomen nudum of Chiltanabaluchi Shakila-Mushtaq & Akbar, 1995.
Chiltana baluchi Shakila-Mushtaq & Akbar, 1995: 374, figs 1–12.

##### Type material examined.

Holotype ♂ of *Chiltanabaluchi* Shakila-Mushtaq & Akbar, Pakistan: (1) [red round label]; (2) Loc. Chiltan Muslim, Host. wild mint, Date. 6.vii.[19]64, Coll. S.M. Khan (ZMUK). Allotype ♀ *Chiltanabaluchi* Shakila-Mushtaq & Akbar, Pakistan: (1) Loc. Chiltan Muolnig, Host. wild mint, Date. 6.vii.[19]64, Coll. S.M. Khan; (2) Dictyophara chiltanii [red written label] (ZMUK).

##### Distribution.

So far only known from Chiltan, Balochistan, Pakistan.

## Discussions

*Chiltana* was firstly described and illustrated as new genus by Shakila-Mushtaq in her Ph.D. thesis which was produced in 1984 (not 1989 as cited by Shakila-Mushtaq in her papers, e.g., Shakila-Mushtaq 1994, Shakila-Mushtaq and Akbar 1995). According to the printed fourth edition of the International Code of Zoological Nomenclature (ICZN 1999), the works, such as a Ph.D. thesis, could be regarded as published if they comply with the requirements of Article 8 and are not excluded by the provisions of Article 9. Shakila-Mushtaq’s thesis satisfied the criteria of Article 8.1, which it was issued for the purpose of providing a public and permanent scientific record (Article 8.1.1.), obtainable when first issued (Article 8.1.2.), and produced in an edition containing simultaneously obtainable copies by a method that assures numerous identical and durable copies (Article 8.1.3.). This thesis also did not provide a statement that the names and acts might be disclaimed (Articles 8.2. and 8.3.), and it was produced before 1986 by a printing method then conventional, i.e., printing on paper (Article 8.4.). This thesis might be considered a published work, and all the names and nomenclatural acts within it might be available under the framework of the Code (ICZN 1999).

However, the International Commission on Zoological Nomenclature (ICZN) has voted in favour of a revised version of the amendment to the Code that was proposed in 2008. The purpose of the amendment is to expand and refine the methods of publication allowed by the Code, particularly in relation to electronic publication. The revised version for the fourth edition of the Code, including the amendments to Articles 8, 9, 10, 21 and 78, with effect from 1 January 2012, has been available online until the fifth edition of the Code is published (ICZN online). A new Article 9.12. has been added in the online version of the Code, which says “facsimiles or reproductions obtained on demand of an unpublished work, even if previously deposited in a library or other archive” do not constitute published work (ICZN online). An example helps to explain this article: “A Ph.D. thesis that was distributed only to members of the student’s thesis committee is listed for sale in the catalogue of a print-on-demand publisher. The print-on-demand work is a reproduction of the thesis. Because the thesis was an unpublished work in its original form, it remains unpublished” (ICZN online). Therefore, according to Article 9.12., we suggest that the Ph.D. thesis of Shakila-Mushtaq (1984) does not constitute published work, and the names in the thesis are regarded as nomina nuda.

Shakila-Mushtaq and Akbar (1995) later described and illustrated *Chiltana* in a published work. We herein suggest that the original authors of *Chiltana* are Shakila-Mushtaq and Akbar, and the date to be adopted is 1995 based on the published work of Shakila-Mushtaq and Akbar (1995).

## Supplementary Material

XML Treatment for Chiltana

XML Treatment for Chiltana acarinata

XML Treatment for Chiltana baluchi
